# Fully Solution-Processed Flexible Organic Thin Film Transistor Arrays with High Mobility and Exceptional Uniformity

**DOI:** 10.1038/srep03947

**Published:** 2014-02-04

**Authors:** Kenjiro Fukuda, Yasunori Takeda, Makoto Mizukami, Daisuke Kumaki, Shizuo Tokito

**Affiliations:** 1 Research Center for Organic Electronics (ROEL), Graduate School of Science and Engineering, Yamagata University, 4-3-16, Jonan, Yonezawa, Yamagata, 992-8510, Japan; 2 Innovation Center for Organic Electronics (INOEL), Graduate School of Science and Engineering, Yamagata University, 1-808-48, Arcadia, Yonezawa, Yamagata, 992-0119, Japan

## Abstract

Printing fully solution-processed organic electronic devices may potentially revolutionize production of flexible electronics for various applications. However, difficulties in forming thin, flat, uniform films through printing techniques have been responsible for poor device performance and low yields. Here, we report on fully solution-processed organic thin-film transistor (TFT) arrays with greatly improved performance and yields, achieved by layering solution-processable materials such as silver nanoparticle inks, organic semiconductors, and insulating polymers on thin plastic films. A treatment layer improves carrier injection between the source/drain electrodes and the semiconducting layer and dramatically reduces contact resistance. Furthermore, an organic semiconductor with large-crystal grains results in TFT devices with shorter channel lengths and higher field-effect mobilities. We obtained mobilities of over 1.2 cm^2^ V^−1^ s^−1^ in TFT devices with channel lengths shorter than 20 μm. By combining these fabrication techniques, we built highly uniform organic TFT arrays with average mobility levels as high as 0.80 cm^2^ V^−1^ s^−1^ and ideal threshold voltages of 0 V. These results represent major progress in the fabrication of fully solution-processed organic TFT device arrays.

Printed electronics has garnered significant attention from research and industry because the pairing of conductive, insulating, and semiconducting materials with printing technologies enables one to make thin, lightweight and low-cost electronic devices and systems^1^,^2^. Organic semiconductors are particularly suitable for printed electronics because they can be processed in solution^3^,^4^,^5^. Moreover, several promising solution-processable organic semiconductor materials have recently been reported^6^,^7^,^8^. Organic materials possess intrinsic mechanical flexibility because of their loose Van der Waals bonding between organic molecules, and they make durable flexible organic devices feasible^9^,^10^,^11^. In particular, flexible thin-film transistor (TFT) devices have recently been developed that have good electrical performance^6^,^7^,^8^, low operating voltages^12^,^13^,^14^, and operational stability^15^,^16^,^17^.

Several novel applications using organic TFT devices or circuits have been developed for purposes such as flexible displays^18^, RFID tags^19^, sensors^20^,^21^, and actuators^22^. These devices have generally been fabricated using vacuum evaporation and photolithography; these mature processes are high resolution, repeatable, and uniform. Yet there are only a few reports on fully printed organic circuits or devices^4^,^23^,^24^,^25^,^26^,^27^,^28^, and wide disparities exist in resolution, electrical performance, and device yield. There is also a wide variability in these device parameters in comparison with devices made using photolithographic processes.

When printed ink dries on the surface of a substrate, the solute is transported from the center to the edge, and the resulting solute film forms a non-uniform ring-like profile, a phenomenon known as the “coffee ring effect^29^”. This effect makes it difficult for fully solution-processed organic electronic devices to be fabricated with high yields or operate at low voltages and with small variations in electrical performance. In addition, printed layers for use in electronic devices have typically possessed deficiencies, such as low conductivities^30^, work functions that deviate from their bulk values^31^,^32^, and rough or porous surfaces^33^. These problems point to a need for comprehensive studies to be done before fully solution-processed organic electronic devices can be commercialized.

In this study, we fabricated an array of fully solution-processed organic TFT devices on flexible plastic substrates and obtained excellent electrical performance and high yields. The use of profile-controlled printed gate electrodes resulted in TFT devices with a very high yield rate (99%) and relatively low operating voltage (20 V). In addition, the use of a source-drain modification layer improved the contact between the electrodes and the semiconducting layers. The resulting TFT devices exhibited mobilities that exceeded 1.0 cm^2^ V^−1^ s^−1^ in ambient air and a low contact resistance (1.8 kΩcm) at gate-source voltage of 20 V. The high yields enabled us to conduct statistical analyses of many solution-processed organic TFT devices produced on the same flexible substrate. The analysis revealed a normal distribution in electrical performance, and this information can be used to simplify the circuit design for fully solution-processed organic electronics.

## Results and discussion

### Fabrication of organic TFT device arrays on plastic films

**Figure 1:**
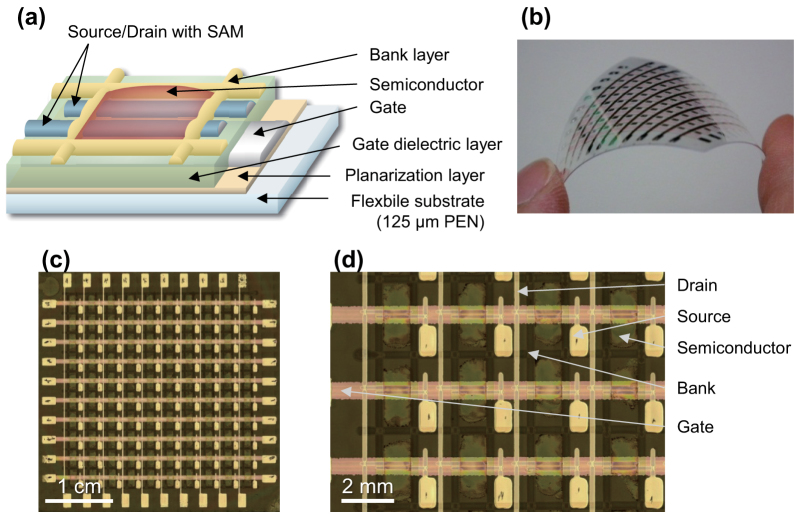
Fully solution-processed organic thin-film transistors on flexible substrates. (a) Schematic cross-section of the TFTs. (b) Photograph of a 10 × 10 TFT array on a flexible PEN substrate. (c) and (d) Optical microscope images of the TFT array.

Fully solution-processed organic TFT devices were fabricated in a 10 × 10 array on 125-μm-thick polyethylene naphthalate (PEN) films with a maximum process temperature of 150°C. Figure 1 shows a schematic illustration and photographs of the TFT device. New materials developed by Merck were employed for the organic semiconductor, gate dielectric, and electrode treatment layers^34^,^35^,^36^. For the electrodes, two formulations of silver nanoparticle ink were used, one for the gate electrodes, in order to form flat surface profiles, the other for the source/drain electrodes, to improve carrier injection into the semiconducting layer. Apart from these materials, cross-linked poly-4-vinylphenol (PVP) and fluoropolymer were used for the planarization layer and bank layer, respectively. An ink-jet printing system and dispenser equipment were used to pattern these materials.

To reduce their surface roughness, the PEN substrates were coated with a 80-nm-thick cross-linked PVP layer, which was deposited using a spin-coating process and cured at a temperature of 150°C. The surface of the cross-linked PVP layer was treated using an oxygen plasma to change the surface wettability. Silver nanoparticle ink was patterned with an inkjet printer onto the cross-linked PVP layers to form the gate electrodes. Following printing, the substrates were stored under controlled temperature and relative humidity conditions in order to planarize the printed electrodes^37^. After the drying process, the substrates were heated at 140°C for 1 hour to sinter the silver nanoparticles. The fabricated silver gate electrodes had uniform thicknesses of about 100 nm. After forming these electrodes, a solution of dielectric polymer materials was spin-coated to form 600-nm-thick gate dielectric layers. After spin coating the dielectric layers, the substrates were heated at 120°C for 1 min on a hotplate, and then cross-linked by using a UV treatment. A separate silver nanoparticle ink was then patterned using an inkjet printer to form the source/drain electrodes, which defined the TFT device geometries. The source/drain electrodes were treated using a self-assembled monolayer (SAM), which was prepared by immersing the substrate for 1 min. 200-nm-thick fluoropolymer bank layers were then printed by using dispenser equipment. The bank layers were used for separation of the semiconducting layers between devices. Finally, a p-type organic semiconducting layer was deposited using dispenser equipment into the area defined by the bank layer, which was then baked at 100°C for 1 min on a hotplate. The ionization potential of the layer used in this study was 5.4 eV. We fabricated several individual TFT devices, which had differing channel widths and lengths. The channel width and length in the array were 1061 ± 8 and 22 ± 5 μm, respectively (see supporting figure S3). The details of the processing can be found in the methodologies section.

### Organic TFT device characterization

**Figure 2:**
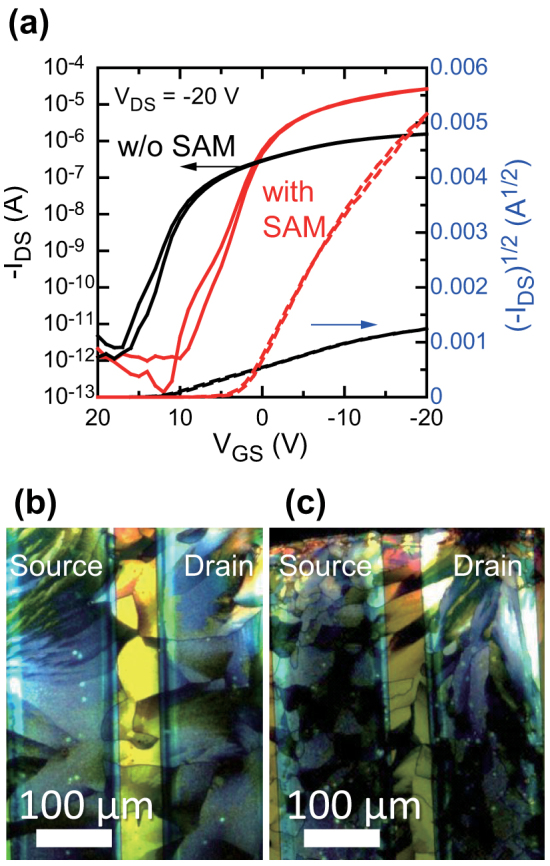
Effect of source-drain electrode modification by SAM treatment on transistor characteristics. (a) Transfer characteristics of fabricated TFTs. The black lines represent the transfer curve for the device without the SAM treatment, and the red lines those with the SAM treatment. Both transistors had almost the same W/L ratio (~50). (b) Polarization microscope images of channel region of fabricated TFTs with untreated and (c) with treated electrodes.

Figure 2a shows the transfer characteristics of the fabricated TFTs, having the same *W/L* ratio of 50, with and without applying a SAM treatment to the source-drain electrodes. The SAM modification process improved the transistor electrical characteristics dramatically, whereby on-current increased from 1.6 μA to 27 μA and the estimated mobility the in saturation regime increased from 0.02 cm^2^ V^−1^ s^−1^ to 0.9 cm^2^ V^−1^ s^−1^. We also observed the crystallinity of semiconducting layer between source/drain electrodes with a polarization microscope, as shown in Figure 2b. Both devices had nearly identical crystalline domains, even though there were large differences in mobility between the devices with the SAM treatment and those without it. The experimental values summarized in Table 1 are the work functions of the treated and untreated silver electrodes measured by using photoemission spectroscopy and their surface energies estimated with the Owens-Wendt method. The work function changed from 4.7 eV before the SAM treatment to 5.3 eV after, but the surface energy did not change.

The carrier injection barrier at the interface between the metal and organic material layers is commonly described by conventional metal-to-semiconductor contact mechanisms^38^. The addition of a carrier injection layer to reduce the energy barrier between the organic semiconducting layer and source/drain electrodes has been well studied^38^,^39^,^40^,^41^. Besides the injection layer, the crystallinity of the semiconducting layer affects the electrical performance of organic TFT devices, especially those based on solution-processed semiconducting layers^6^,^7^,^42^. In this study, it was found that the SAM treatment affected neither the surface energy of the source/drain electrodes nor the crystalline structure of the semiconducting layer. However, the SAM modification process did change the work function of those electrodes and, as a result, the energy gap between the work function of the source-drain electrodes and the ionization potential of the organic semiconducting layer decreased from 0.7 eV to 0.1 eV. This matching between the electrode work function and the semiconducting layer ionization potential resulted in significant improvements in the electrical performance of our TFT devices. Therefore, it can be said that the SAM-treated source/drain electrodes had reduced carrier injection and lower the contact resistance relative to the non-treated electrodes. Furthermore, it is known that the work functions of printed electrodes are sensitive to the surrounding ambient or process conditions^31^,^32^. These results strongly suggest that printed electrodes should be treated properly in order to reduce the energy barrier between the organic semiconducting layer and source/drain electrodes.

**Figure 3:**
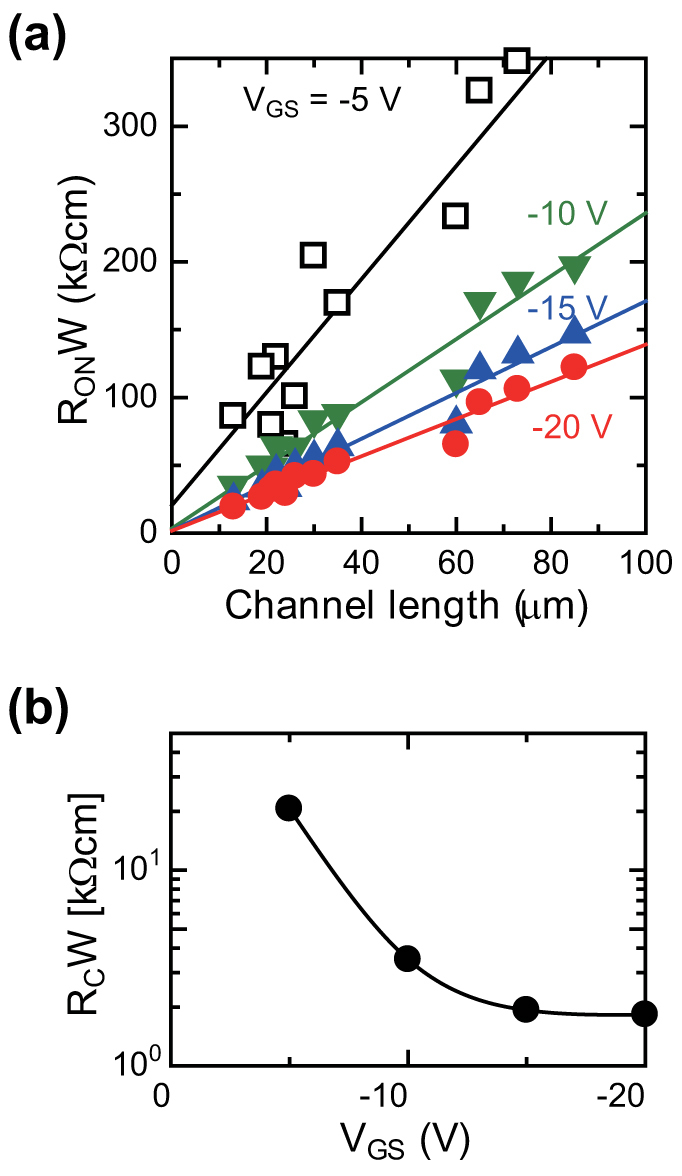
Estimation of contact resistance. The contact resistance of the TFT devices with treated source-drain electrodes were estimated by using the transfer-line method. (a) Channel width-normalized total on-resistance (*R_ON_*) as a function of channel length. (b) Width-normalized contact resistance as a function of gate-source voltage (*V_GS_*).

We estimated the contact resistance (*R_C_*) of the fabricated TFT devices using a transfer-line method^43^. The fabricated TFT devices exhibited disparities in their mobility levels; therefore, we used only the TFT devices with mobilities of more than 0.8 cm^2^ V^−1^s^−1^ for our estimation of *R_C_*. Figure 3a plots the channel width-normalized total on-resistance (*R_ON_*) as a function of channel length. *R_C_* was obtained by extrapolating the linear fit to a channel length of zero and plotted as a function of gate-source voltage (*V_GS_*) (Fig. 3b). *R_C_* decreases with increasing gate-source voltage, likely due to an increase in carrier density in the channel and near the contacts. *R_C_* decreased to a value as low as 1.83 kΩcm, a remarkably low contact resistance value for fully solution-processed organic TFT devices, which is attributed to there being a low energy barrier between the printed organic semiconducting layer and source/drain electrodes.

### Dependence of TFT electrical characteristics on channel length

**Figure 4:**
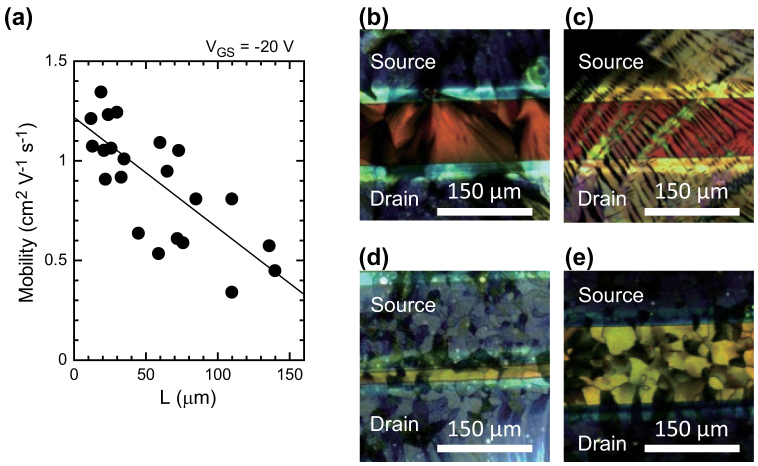
Dependence of field-effect mobility in saturation region on channel-length. (a) Mobility as a function of channel length. The black dots represent the experimental data, and the solid line is a fitted curve. (b)–(e) Polarization microscope images of the channel region of fabricated TFTs. The channel length and mobility are as follows: (b) 110 μm and 0.81 cm^2^ V^−1^ s^−1^, (c), 110 μm and 0.34 cm^2^ V^−1^ s^−1^, (d) 20 μm and 1.23 cm^2^ V^−1^ s^−1^, (e) 140 μm and 0.45 cm^2^ V^−1^ s^−1^.

Next, we evaluated the dependence of the electrical performance of fabricated TFT devices on the channel length. Figure 4a shows the field effect mobility of the TFT devices with SAM-treated source/drain electrodes in the saturation region as a function of channel length. These plots clearly show a correlation between the channel length and mobility, such that mobility decreases almost linearly with channel length between 10 μm and 150 μm. A fitted regression line has negative slope and a correlation coefficient *R^2^* of 0.57. In general, the mobility of organic TFTs decreases as the channel length decreases below 20 μm because of large contact resistance values^44^,^45^. Gundlach reported a similar channel length dependence in organic TFTs with solution-processed semiconductors and discussed the relation between such tendencies and the crystal structure of the semiconducting layers^42^. Accordingly, we observed the channel region of the TFTs using the polarizing microscope and estimated the degree of crystallinity in the channel layer. Four images of the channel region of TFTs with different mobilities and channel lengths are shown in Figures 4b–e. First, we compared the two TFTs with the same channel length (110 μm), but with different mobilities. The TFT with a higher mobility (0.81 cm^2^ V^−1^ s^−1^) had semiconducting layers with large crystalline domains, as shown in Figure 4b. Here, a single crystalline domain covered the entire length of the channel layer. In contrast, the TFT with the lower mobility (0.34 cm^2^ V^−1^ s^−1^) exhibited needle-like crystalline structures (Fig. 4c). Here, the many grain boundaries between the source and drain electrodes resulted in relatively low mobility levels.

Next, we compared the other two TFTs with different channel lengths. Figure 4d shows an image of TFTs with a channel length of 24 μm and mobility of 1.24 cm^2^ V^−1^ s^−1^, and Figure 4e shows an image of TFTs with a channel length of 140 μm and mobility of 0.45 cm^2^ V^−1^ s^−1^. As can be seen in the polarizing microscope images, these two TFTs had similar crystalline layers, each with domain sizes of approximately 30 μm. The relatively large domain sizes produced single-domain crystalline layers that extended across the short channel length region between the source and drain electrodes. However, there were several grain boundaries between the source and drain electrodes for TFTs with wider channel lengths, which would inhibit carrier transport between these electrodes. These results are consistent with previous studies^46^,^47^,^48^. These observations suggest that printed organic TFT devices with a short channel length may be able to have a high mobility and excellent frequency response if the source-drain electrodes can be appropriately modified such that the single crystalline domain within the semiconducting layer can cover the entire channel region. Moreover, in terms of electrical performance, these short-channel TFT devices are the best among fully solution-processed organic TFT devices reported so far^49^,^50^ and comparable with those using non-printed source/drain electrodes. A non-printed device made from the same semiconductor material, evaporated Al gate and Au source/drain electrodes, and the same treatment processes operated with a mobility of 1.7 cm^2^ V^−1^s^−1^ (see supporting figure S11). This comparison in mobility between fully printed and non-printed devices indicates that our fully printed TFT devices get the best possible electrical performance from the given semiconductor material.

### Mechanical and operation stability

**Figure 5:**
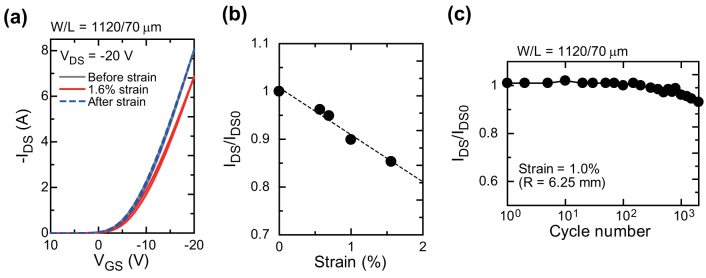
Mechanical flexibility of fully solution-processed organic TFT devices. (a) Transfer characteristics of pentacene-based TFT device before (gray solid line), during (red solid line), and after (blue dashed line) application of 1.6% tensile strain. (b) Change in current as a function of tensile strain. The on-current was normalized by its initial value. (c) Mechanical durability during repeated 1.0% tensile strain and relaxation. Even after 1000 cycles, the change in on-current was less than 5%.

To demonstrate the flexibility of the fabricated devices, tensile strains were applied to the organic TFT devices. The strains were parallel to the source-drain current paths, and the electrical performances of the devices were evaluated before, during, and after the application of strain. We applied bending strains with radii (R) ranging from 11 mm to 4 mm, corresponding to an induced surface strain from 0.57% to 1.6%. The electrical behavior of the devices was characterized during the systematic application of tensile strain. Figure 5a shows the transfer characteristics of a device with a channel length of 60 μm. The gate voltage V_GS_ was swept from 10 to −20 V while V_DS_ was kept at −20 V. In agreement with previous reports, this fully printed device exhibited a decrease in saturation on-current I_DS_ upon the application of tensile strain^51^,^52^,^53^. The transfer characteristics completely returned to the initial state after the tensile strain. The change in the on-current was −15% at 1.6% tensile strain. The normalized on-current is plotted as a function of tensile strain in Figure 5b. The current change was linearly proportional to the applied surface strain. This indicates that the origin of the current change in fully printed organic TFT devices under strain is essentially the same as that of the evaporated organic TFT devices^51^,^52^. A stress cycle was also applied to the TFT devices. First, the device was bent upwards from a flat state into one with a 6.25 mm radius which corresponded to a 1.0% tensile strain, and was immediately released to unbend back into the flat state. After that, the FET was bent downwards to R = 6.25 mm and released. This bending cycle was repeated at a rate of 30 times a minute. Figure 5c plots the normalized *I_DS_* as a function of the number of outward bending cycles. Even after 1000 full cycles, the change in on-current amounted to less than 5%. This excellent flexibility is attributed to the strong fusion of the electrodes with the underlying dielectric surfaces and semiconducting layers. These results show the feasibility of using fully printed electronic devices in flexible systems.

**Figure 6:**
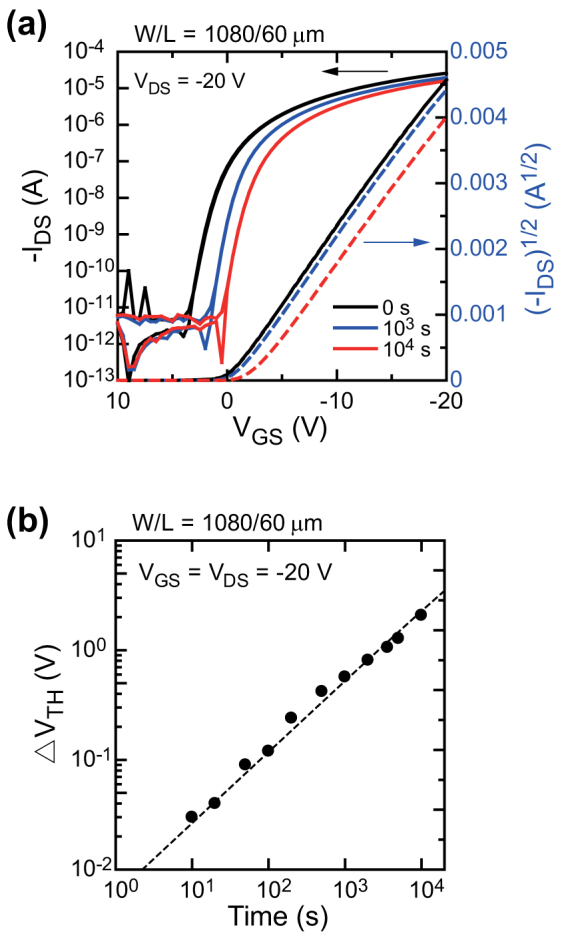
Operational stability. (a) Transfer characteristics of the device taken before and after continuous application of bias voltage (*V_GS_ = V_DS_* = −20 V). The black lines represent the results before applying bias stress, the blue lines represent those after applying bias stress for 10^3^ s, and the red lines represent those after applying bias stress for10^4^ s. (b) Threshold voltage shift (*ΔV_TH_*) as a function of time while applying continuous bias voltages (*V_GS_ = V_DS_* = −20 V). The black circles represent the experimental results, and the red dashed line is the fitted curve.

We also evaluated the operational stability of the devices. We estimated the change in threshold voltage (*ΔV_TH_*) as the devices were stressed with constant bias voltages (*V_GS_* = *V_DS_* = −20 V) from their transfer characteristics. Figure 6a shows the changes in transfer characteristics before and after stressing the devices for 10^3^ and 10^4^ s in ambient air. Though the transfer characteristics shifted slightly in the negative direction, the other key electrical parameters such as on/off ratio and field-effect mobility did not change with the negative gate bias stress. *ΔV_TH_* as a function of stress time is plotted in Figure 6b. *ΔV_TH_* was 1.1 V at 10^3^ s and 2.1 V at 10^4^ s. This level of operational stability is comparable with evaporated organic TFT devices^54^ and could be further improved by using appropriate suppression techniques such as surface treatment of the dielectric layers and/or a combination of dielectric layers^17^,^55^.

### Organic TFT array characterization

**Figure 7:**
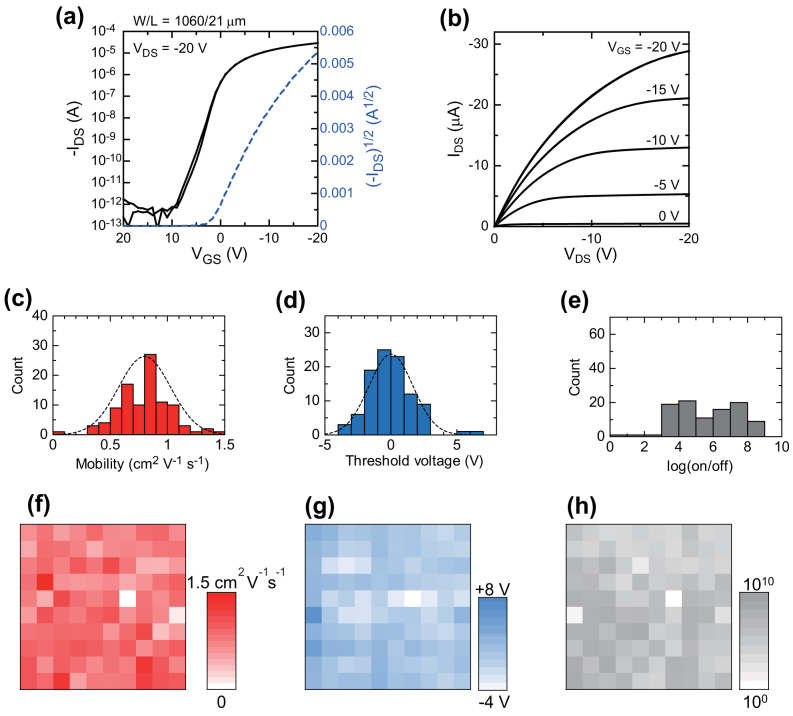
Electrical characteristics of 10 × 10 TFT array. (a) Transfer characteristics at *V_DS_* = −20 V. (b) Output characteristics at various gate voltages *V_GS_*. Distribution of mobilities (c), threshold voltages (d), and on/off ratios (e) as measured from 99 transistors in the array. Average mobility is 0.80 ± 0.23 cm^2^ V^−1^ s^−1^, and average threshold voltage is 0.01 ± 1.67 V. Dispersion of mobilities (f), threshold voltages (g), and on/off ratios (h).

A fully solution-processed organic TFT array (10 × 10 layout) was fabricated on a 40 mm × 40 mm PEN film, and 99% of the fabricated TFT devices functioned well. The high yield was largely the result of fully solution-processed gate electrodes with flat profiles^37^, and it enabled a statistical evaluation to be carried out on the devices within the TFT array. The transfer characteristics and output characteristics of a typical device in the array are shown in Figures 7a and 7b. The transistor exhibited excellent electrical performance with no hysteresis in either the transfer or output characteristics. The output characteristics showed good linearity in the low source-drain voltage region (*V_DS_* < 5 V), which indicates there was good ohmic contact between the source/drain electrodes and the semiconducting layer. Figures 7c–e plot the distributions of various transistor-performance values. The mobility in the saturation regime was on average 0.80 ± 0.23 cm^2^ V^−1^ s^−1^, and the maximum value was 1.5 cm^2^ V^−1^ s^−1^. The average threshold voltage was 0.0 ± 1.7 V. These variations are comparable with those of organic TFT devices with thermally evaporated metal source/drain electrodes on glass substrates^6^,^56^. The on/off current ratios ranged from 10^4^ to 10^9^, and the primary reason for this wide discrepancy is variations in off-current. There are two main causes of the off-current variations. One is the properties of the gate dielectric layer. The printed gate layers had a relatively large surface roughness of 3 nm and a thickness of 100 nm; as a result, some TFT devices exhibited large gate leakage currents, and that led to larger off-currents. The second reason is the crystalline structure of the semiconducting layer. Thicker semiconducting layers tend to exhibit larger off-current levels, resulting in smaller on/off current ratios. These variations in on/off current could be suppressed by using improved fabrication techniques for the gate dielectric and semiconducting layers. Figures 7f–h plot the electrical characteristics in two dimensions with the intensity of the color representing the measured performances. These plots indicate that the variations in device performance appear randomly on the same substrates.

The remaining variations in performance of devices within the array were caused by differences in the crystallinity of the semiconducting layer. Indeed, the crystallinity varied from device to device (see supporting Figure S2), and this indicates that the crystal growth of the solution-processed organic semiconducting layer should be more precisely controlled in order to achieve uniform electrical characteristics. In addition, deviations in critical device dimensions such as the channel length and channel width were responsible for some of the variation. Both the channel width and length followed statistically normal distributions (see supporting Figure S3). Interestingly, mobility and threshold voltage also followed normal distribution curves, as shown in Figures 7c and 7d, despite the variations in the crystallinity of the semiconducting layer. These variations (standard deviations) in performance across TFT devices could be suppressed by better controlling the device dimensions. The high performance and relatively high uniformity of our fully solution-processed organic TFT device arrays demonstrate the feasibility of using them in the design of reliable digital logic circuits.

## Conclusion

We fabricated organic TFT arrays on plastic film substrates by using solution processes performed on ink-jet printer and dispenser equipment. The results of our study demonstrate the tremendous potential that exists for printed electronics. By making improvements to the materials, modifications to the design of the electrodes, and forming uniform and flat electrodes, we can fabricate fully solution-processed organic TFT arrays with a high yield and excellent electrical characteristics at low operating voltages. Our results show a clear correlation between the semiconductor layer's crystallinity and the channel length with the electrical performance of the organic TFTs. The statistical analysis of the TFT array obtained useful data for designing organic TFT devices and circuits for practical applications. These fabrication methods will help enable novel low-temperature, low-cost, large-area manufacturing of printed electronics.

## Methods

### Device fabrication

A 125-μm-thick PEN film (Teijin DuPont Films, Teonex®) was used as the flexible substrate, and cross-linked PVP was used as the planarization layer. PVP (Mw ~ 25000, Sigma Aldrich Co.) and poly(melamine-co-formaldehyde) (Mn ~ 432, 84 wt%, Sigma Aldrich Co.) as a cross-linking agent were mixed in propylene glycol monomethyl ether acetate (PGMEA). The PEN substrates were coated with an 80-nm-thick cross-linked PVP layer to reduce the surface roughness, which was deposited by spin-coating and cured at a temperature of 150°C. The surface of cross-linked PVP layer was then treated for 1 min with oxygen plasma (plasma power of 100 W) to change the surface wettability. Silver (Ag) nanoparticle ink in aqueous solvent (DIC Corp., JAGLT-01) was patterned with an inkjet printer (Fujifilm Dimatix, model DMP2800) onto the cross-linked PVP layers by using a print head with 10 pl nozzles to form the gate electrodes. The Ag nanoparticle ink was printed using a customized waveform, and the droplets were deposited with a dot-to-dot spacing of 60 μm. During the inkjet patterning process, the substrate temperature was maintained at 30°C. After printing, the substrates were stored for 30 min in an environmental test chamber (ESPEC, model SH-221) in which the temperature was held at 30°C and relative humidity was held at 95% in order to planarize the electrodes^37^. After the drying process, the substrates were heated at 140°C for 1 hour to sinter the silver nanoparticles. The fabricated silver gate electrodes had a uniform thickness of about 100 nm. After forming the electrodes, a solution of polymer dielectrics (Merck, lisicon® D207) was spin-coated to form 600-nm-thick gate dielectric layers. This dielectric material has a relative permittivity of 2.8, which corresponds to a capacitance of 4.1 nF cm^−2^. After spin coating of the dielectrics, the substrates were heated at 120°C for 1 min on a hotplate, and then cross-linked by using a UV treatment (Ushio Inc., model UX-3). The wavelength of the UV radiation was 365 nm, and the dosage was 2650 mJ cm^−2^. Silver nanoparticle ink in a tetradecane-based solvent (NPS-JL, Harima Chemicals) was patterned with an inkjet printer to form the source/drain electrodes. The droplets were deposited with a dot-to-dot spacing of 60 μm. The TFT device geometry was defined by the patterning data of inkjet printing. A self-assembled monolayer (SAM) treatment for source/drain electrodes (Merck, lisicon® M001) was prepared by dipping the substrate into a solution of propanol for 1 min. Then, a solution of fluoropolymer (DuPont™, Teflon® AF 1600) in Fluorinert (3M™ FC-43) was used as a bank layer and printed by using dispenser equipment (Musashi Engineering, Image Master 350 PC) that included a three-axis table and an air dispenser, both of which were computer-controlled to dispense and pattern the solution. The 200-nm-thick fluoropolymer bank layers were printed at a patterning speed of 20 mm/s and discharge pressure of 7 kPa. The substrate and nozzle temperature were maintained at 30°C during the dispenser patterning process. After printing of the bank layer, the substrates were stored in air ambient for 10 min to evaporate (dry) the solvent from the fluoropolymer solution. Finally, a mesitylene-based formulation of a soluble small-molecule organic semiconducting layer (Merck, lisicon® S1200) with a deep ionization potential of 5.4 eV was printed into the area defined by the bank layer by using dispenser equipment at a patterning speed of 20 mm/s and discharge pressure of 1 kPa. The substrate and nozzle temperature were maintained at 30°C during the dispenser patterning process. After patterning the semiconducting layer, the substrates were baked at 100°C for 1 min on a hotplate.

### Device characterization

The electrical characteristics of the fabricated capacitors and TFT devices were measured by using a semiconductor parameter analyzer (Keithley, model 4200-SCS). All electrical measurements on the organic TFT devices were carried out in air ambient. The surfaces of the fabricated devices were observed using a laser microscope (Olympus, model OLS-4000) and polarization microscope (Nikon ECLIPSE ME600). The work function of the source/drain electrodes was measured using photoemission spectroscopy (Riken Keiki Co., Ltd., model AC-3). The surface energy of the treated and un-treated source/drain electrodes were estimated by using the Owens-Wendt method and a contact angle meter (Attension, model Theta T200-Basic)^57^.

## Author Contributions

K.F., Y.T. and M.M. carried out experimental work and data analysis. K.F., D.K. and S.T. conceptualized the research and wrote the manuscript.

## Supplementary Material

Supplementary Information Supplementary information

## Figures and Tables

**Table 1:** Measured work function, surface energy, and transistor characteristics of treated and untreated silver electrodes

	Work function (eV)	Surface energy (mN m^−1^)	μ (cm^2^ V^−1^ s^−1^)	On-current (μA)
With SAM	5.3	30.7	0.9	27
Without SAM	4.7	33.1	0.02	1.6

## References

[b1] Perelaer J. *et al.* Printed electronics: the challenges involved in printing devices, interconnects, and contacts based on inorganic materials. J. Mater. Chem. 20, 8446–8453 (2010).

[b2] Ahn B. *et al.* Omnidirectional Printing of Flexible, Stretchable, and Spanning Silver Microelectrodes. Science 323, 1590–1593 (2009). 19213878 10.1126/science.1168375

[b3] Yan H. *et al.* A high-mobility electron-transporting polymer for printed transistors. Nature 457, 679–686 (2009). 19158674 10.1038/nature07727

[b4] Gili E., Caironi M. & Sirringhaus H. Organic integrated complementary inverters with ink-jet printed source/drain electrodes and sub-micron channels. Appl. Phys. Lett. 100, 123303 (2012).

[b5] Kang H., Kitsomboonloha R., Jang J. & Subramanian V. High-Performance Printed Transistors Realized Using Femtoliter Gravure-Printed Sub-10 μm Metallic Nanoparticle Patterns and Highly Uniform Polymer Dielectric and Semiconductor Layers. Adv. Mater. 24, 3065–3069 (2012). 22570314 10.1002/adma.201200924

[b6] Minemawari H. *et al.* Inkjet printing of single-crystal films. Nature 475, 364–367 (2011). 21753752 10.1038/nature10313

[b7] Giri G. *et al.* Tuning charge transport in solution-sheared organic semiconductors using lattice strain. Nature 480, 504–509 (2011). 22193105 10.1038/nature10683

[b8] Nakayama K. *et al.* Patternable Solution-Crystallized Organic Transistors with High Charge Carrier Mobility. Adv. Mater. 23, 1626–1629 (2011). 21472790 10.1002/adma.201004387

[b9] Sekitani T. Zschieschang U. Klauk H. & Someya T. Flexible organic transistors and circuits with extreme bending stability. Nature Mater. 9, 1015–1022 (2010). 21057499 10.1038/nmat2896

[b10] Yi H. T., Payne M. M., Anthony J. E. & Podzorov V. Ultra-flexible solution-processed organic field-effect transistors. Nature Commun. 3, 1259 (2012). 23232389 10.1038/ncomms2263

[b11] Kaltenbrunner M. *et al.* Ultrathin and lightweight organic solar cells with high flexibility. Nature Commun. 3, 770 (2012). 22473014 10.1038/ncomms1772 PMC3337988

[b12] Klauk H., Zschieschang U., Pflaum J. & Halik M. Ultralow-power organic complementary circuits. Nature 445, 745–748 (2007). 17301788 10.1038/nature05533

[b13] Cho J. H. *et al.* Printable ion-gel gate dielectrics for low-voltage polymer thin-film transistors on plastic. Nature Mater. 7, 900–906 (2008). 18931674 10.1038/nmat2291

[b14] Yoon M. –H., Facchetti A. & Marks T. J. σ-π molecular dielectric multilayers for low-voltage organic thin-film transistors. Proc. Natl. Acad. Soc. U.S.A. 102, 4678–4682 (2005). 10.1073/pnas.0501027102 PMC555722 15781860

[b15] Yokota T. *et al.* Flexible Low-Voltage Organic Transistors with High Thermal Stability at 250°C. Adv. Mater. 25, 3639–3644 (2013). 23616376 10.1002/adma.201300941

[b16] Kuribara K. *et al.* Organic transistors with high thermal stability for medical applications. Nature Commun. 3, 723 (2012). 22395614 10.1038/ncomms1721

[b17] Fukuda K. Suzuki T. Kobayashi T., Kumaki D. & Tokito S. Suppression of threshold voltage shifts in organic thin-film transistors with bilayer gate dielectrics. Phys. Status Solidi A 210, 839–844 (2013).

[b18] Noda M. *et al.* An OTFT-driven rollable OLED display. J. Soc. Inf. Display 19, 316–322 (2011).

[b19] Myny K. *et al.* Organic RFID transponder chip with data rate compatible with electronic product coding. Org. Electron. 11, 1176–1179 (2010).

[b20] Someya T. *et al.* Conformable, flexible, large-area networks of pressure and thermal sensors with organic transistor active matrixes. Proc. Natl. Acad. U.S.A. 102, 12321–12325 (2005). 10.1073/pnas.0502392102 PMC1187825 16107541

[b21] Kato Y. *et al.* Large-Area Flexible Ultrasonic Imaging System With an Organic Transistor Active Matrix. IEEE Trans. Electron Devices 57, 995–1002 (2010).

[b22] Fukuda K. *et al.* A 4 V Operation, Flexible Braille Display Using Organic Transistors, Carbon Nanotube Actuators, and Organic Static Random-Access Memory. Adv. Funct. Mater. 21, 4019–4027 (2011).

[b23] Tseng H. –Y., Purushothaman B., Anthony J. & Subramanian V. High-speed organic transistors fabricated using a novel hybrid-printing technique. Org. Electron. 12, 1120–1125 (2011).

[b24] Ng T. N. *et al.* Scalable printed electronics: an organic decoder addressing ferroelectric non-volatile memory. Sci. Rep. 2, 585 (2012). 22900143 10.1038/srep00585 PMC3420218

[b25] Schwartz D. E. & Ng T. N. Comparison of Static and Dynamic Printed Organic Shift Registers. IEEE Electron. Device Lett. 34, 271 (2013).

[b26] Suzuki K. *et al.* A 200 ppi All-Printed Organic TFT Backplane for Flexible Electrophoretic Displays. Proc. IDW 09, 1581–1584 (2009).

[b27] Hambsch M., Reuter K., Kempa H. & Hübler A. C. Comparison of fully printed unipolar and complementary organic logic gates. Org. Electron. 13, 1989–1995 (2012).

[b28] Jung M. *et al.* All-Printed and Roll-to-Roll-Printable 13.56-MHz-Operated 1-bit RF Tag on Plastic Foils. IEEE Trans. Electron Devices 57, 571–580 (2010).

[b29] Deegan R. D. *et al.* Capillary flow as the cause of ring stains from dried liquid drops. Nature 389, 827–829 (1997).

[b30] Sirringhaus H. *et al.* High-Resolution Inkjet Printing of All-Polymer Transistor Circuits. Science 290, 2123–2126 (2000). 11118142 10.1126/science.290.5499.2123

[b31] Fukuda K. *et al.* Organic integrated circuits using room-temperature sintered silver nanoparticles as printed electrodes. Org. Electron. 13, 3296–3301 (2012).

[b32] Kim D. Shin H. Xia Y. & Moon J. Heterogeneous Interfacial Properties of Ink-Jet-Printed Silver Nanoparticulate Electrode and Organic Semiconductor. Adv. Mater. 20, 3084–3089 (2008).

[b33] Magdassi S., Grouchko M., Berezin O. & Kamyshny A. Triggering the Sintering of Silver Nanoparticles at Room Temperature. ACS Nano, 4, 1943–1948 (2010). 20373743 10.1021/nn901868t

[b34] Lloyd G. *et al.* Development of Printed, High-Performance Organic Semiconductor Devices. Proc. IDW 10, 469 (2010).

[b35] Jamesn M. *et al.* Printable Organic Thin Film Transistor Backplanes for Mass Produced Displays. SID Int. Symp. Dig. Tec. 43, 422–425 (2012).

[b36] Fujisaki Y. *et al.* Direct patterning of solution-processed organic thin-film transistor by selective control of solution wettability of polymer gate dielectric. Appl. Phys. Lett. 102, 153305 (2013).

[b37] Fukuda K., Sekine T., Kumaki D. & Tokito S. Profile Control of Inkjet Printed Silver Electrodes and Their Application to Organic Transistors. ACS Appl. Mater. Interfaces 5, 3916–3920 (2013). 23547936 10.1021/am400632s

[b38] Kumaki D., Fujisaki Y. & Tokito S. Reduced contact resistance and highly stable operation in polymer thin-film transistor with aqueous MoO_x_ solution contact treatment. Org. Electron. 14, 475–478 (2013).

[b39] Shrotriya V., Li G., Yao Y., Chu C. –W. & Yang Y. Transition metal oxides as the buffer layer for polymer photovoltaic cells. Appl. Phys. Lett. 88, 073508 (2008).

[b40] Ishii H., Sugiyama K., Ito E. & Seki K. Energy Level Alignment and Interfacial Electronic Structures at Organic/Metal and Organic/Organic Interfaces. Adv. Mater. 11, 605–625 (1999).

[b41] Campbell I. H. *et al.* Controlling charge injection in organic electronic devices using self-assembled monolayers. Appl. Phys. Lett. 71, 3528–3530 (1997).

[b42] Gundlach D. J. *et al.* Contact-induced crystallinity for high-performance soluble acene-based transistors and circuits. Nature Mater. 7, 216–221 (2008). 18278050 10.1038/nmat2122

[b43] Klauk H. *et al.* Contact resistance in organic thin film transistors. Solid-State Electron. 47, 297–301(2003).

[b44] Yokota T. *et al.* Low-voltage organic transistor with subfemtoliter inkjet source–drain contacts. MRS Commun. 1, 3–6 (2011).

[b45] Gundlach D. J. *et al.* An experimental study of contact effects in organic thin film transistors. J. Appl. Phys. 100, 024509 (2006).

[b46] Lee S. S. *et al.* Controlling Nucleation and Crystallization in Solution-Processed Organic Semiconductors for Thin-Film Transistors. Adv. Mater. 21, 3605–3609 (2009).

[b47] Horowitz G. & Hajlaoui M. E. Grain size dependent mobility in polycrystalline organic field-effect transistors. Synthetic Met. 122, 185–189 (2001).

[b48] Podzorov V. Organic single crystals: Addressing the fundamentals of organic electronics. MRS bulletin 38, 15–27 (2013).

[b49] Whiting G. L. & Arias A. C. Chemically modified ink-jet printed silver electrodes for organic field-effect transistors. Appl. Phys. Lett. 95, 253302 (2009).

[b50] Zhao Y. *et al.* All-Solution-Processed, High-Performance n-Channel Organic Transistors and Circuits: Toward Low-Cost Ambient Electronics. Adv. Mater. 23, 2448–2453 (2011). 21394796 10.1002/adma.201004588

[b51] Sekitani T. *et al.* Bending experiment on pentacene field-effect transistors on plastic films. Appl. Phys. Lett. 86, 073511 (2005).

[b52] Cosseddu P. *et al.* Continuous tuning of the mechanical sensitivity of Pentacene OTFTs on flexible substrates: From strain sensors to deformable transistors. Org. Electron. 14, 206–211 (2013).

[b53] Fukuda K. *et al.* Strain sensitivity and durability in p-type and n-type organic thin-film transistors with printed silver electrodes. Sci. Rep. 3, 2048; 10.1038/srep02048 (2013). 23788235 10.1038/srep02048 PMC3689172

[b54] Umeda T., Kumaki D. & Tokito S. High air stability of threshold voltage on gate bias stress in pentacene TFTs with a hydroxyl-free and amorphous fluoropolymer as gate insulators. Org. Electron. 9, 545–549 (2008).

[b55] Suemori K. Uemura S. Yoshida M. Hoshino S. & Takada N. Threshold voltage stability of organic field-effect transistors for various chemical species in the insulator surface. Appl. Phys. Lett. 91, 192112 (2007).

[b56] Ikawa M. *et al.* Simple push coating of polymer thin-film transistors. Nature Commun. 3, 1176 (2012). 23132026 10.1038/ncomms2190 PMC3493649

[b57] Owens D. K. & Wendt R. C. Estimation of the surface free energy of polymers. J. Appl. Polym. Sci. 13, 1741–1747 (1969).

