# Supplementary material for: Fully Solution-Processed Flexible Organic Thin Film Transistor Arrays with High Mobility and Exceptional Uniformity
Source: Sci Rep. 2014 Feb 4;4:3947. doi: 10.1038/srep03947 (PMC3912504; doi:10.1038/srep03947)
Supplement: Supplementary Information [file srep03947-s1.pdf]

Supporting Information

Fully Solution-Processed Flexible Organic Thin Film Transistor Arrays with High Mobility and Exceptional Uniformity

Kenjiro Fukuda(*)[1,2], Yasunori Takeda[1,2], Makoto Mizukami[1,3] Daisuke Kumaki[1,2], and Shizuo Tokito[1,2]

[1]Graduate School of Science and Engineering, Yamagata University, 4-3-16 Jonan, Yonezawa, Yamagata, 992-8510, Japan

[2]Research Center for Organic Electronics, Yamagata University

[3]Innovation Center for Organic Electronics, Yamagata University

Address correspondences to fukuda@yz.yamagata-u.ac.jp

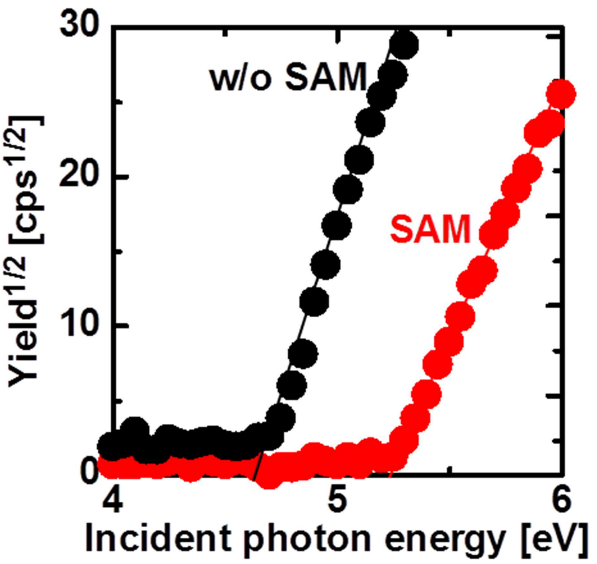

Figure S1. Work function of the printed source/drain electrodes.

Square-root of the yield as a function of incident photon energy from photoemission spectroscopy. The black dots represent the untreated electrodes, and the red dots represent the treated electrodes.

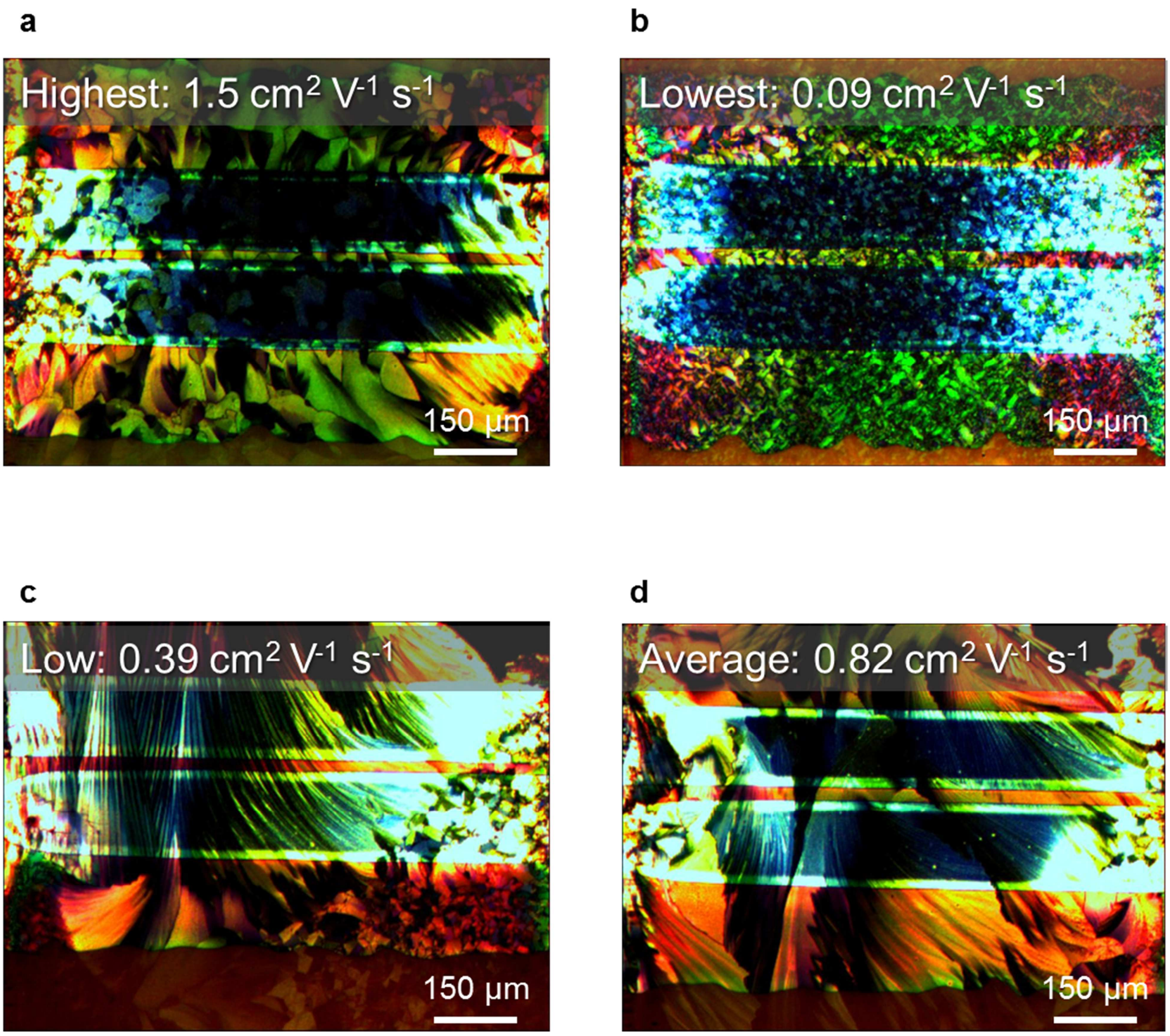

Figure S2. Polarization microscope images of the devices within the TFT array.

The mobility values are as follows: **a,** Highest (1.5 cm^2 V^{-1} s^{-1}), **b,** Lowest (0.09 cm^2 V^{-1} s^{-1}), **c,** Low (0.39 cm^2 V^{-1} s^{-1}) **d,** Average (0.82 cm^2 V^{-1} s^{-1}).

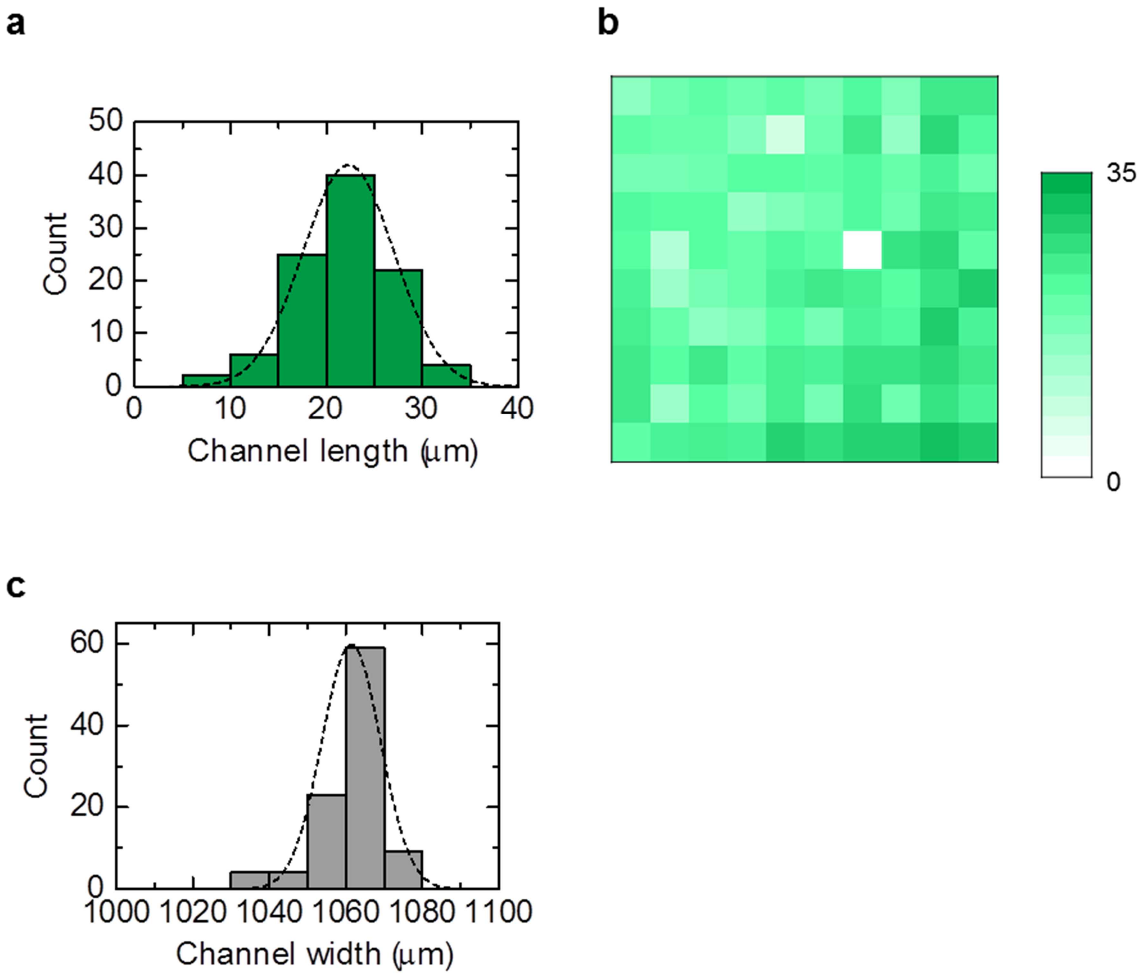

Figure S3. Distribution of TFT geometry.

a, Distribution of channel length for the measured 99 transistors in the array. Average channel length is 22.3 ± 4.8 µm. The black dot line represents the normal distribution. **b,** A two-dimensional plot of channel length dispersion with color intensity. **c**, Distribution of channel width for the measured 99 transistors in the array. Average channel width is 1061 ± 8 µm.

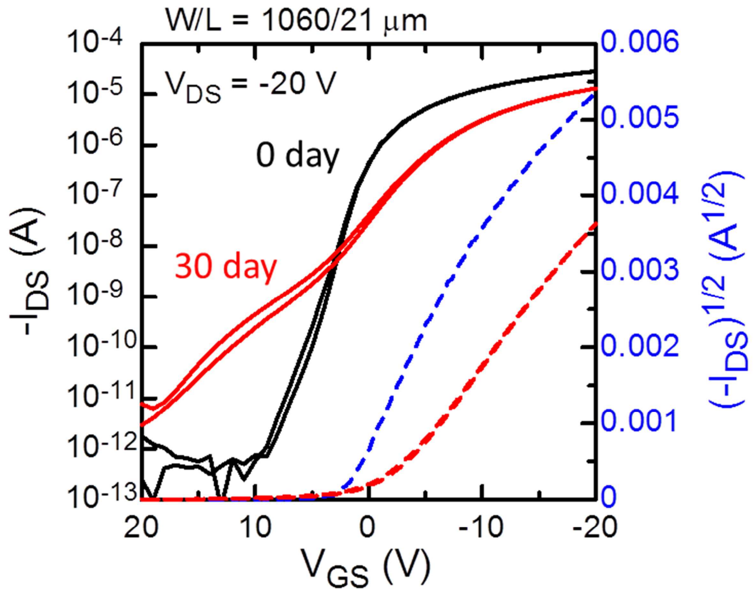

Figure S4. Air stability of the fabricated organic TFT devices.

The fabricated organic TFTs were stored in ambient air conditions with a temperature of about 25 °C and a humidity of about 30%RH for a period of 30 days. The black line represents the transistor characteristics recorded shortly after device fabrication and red line represents that recorded after 30 days in air. The estimated mobility in saturation regime decreased from 1.1 to 0.43 $cm^2\ V^{-1}\ s^{-1}$ and the threshold voltage shifted from 2.0 to –1.7 V after 30 days.

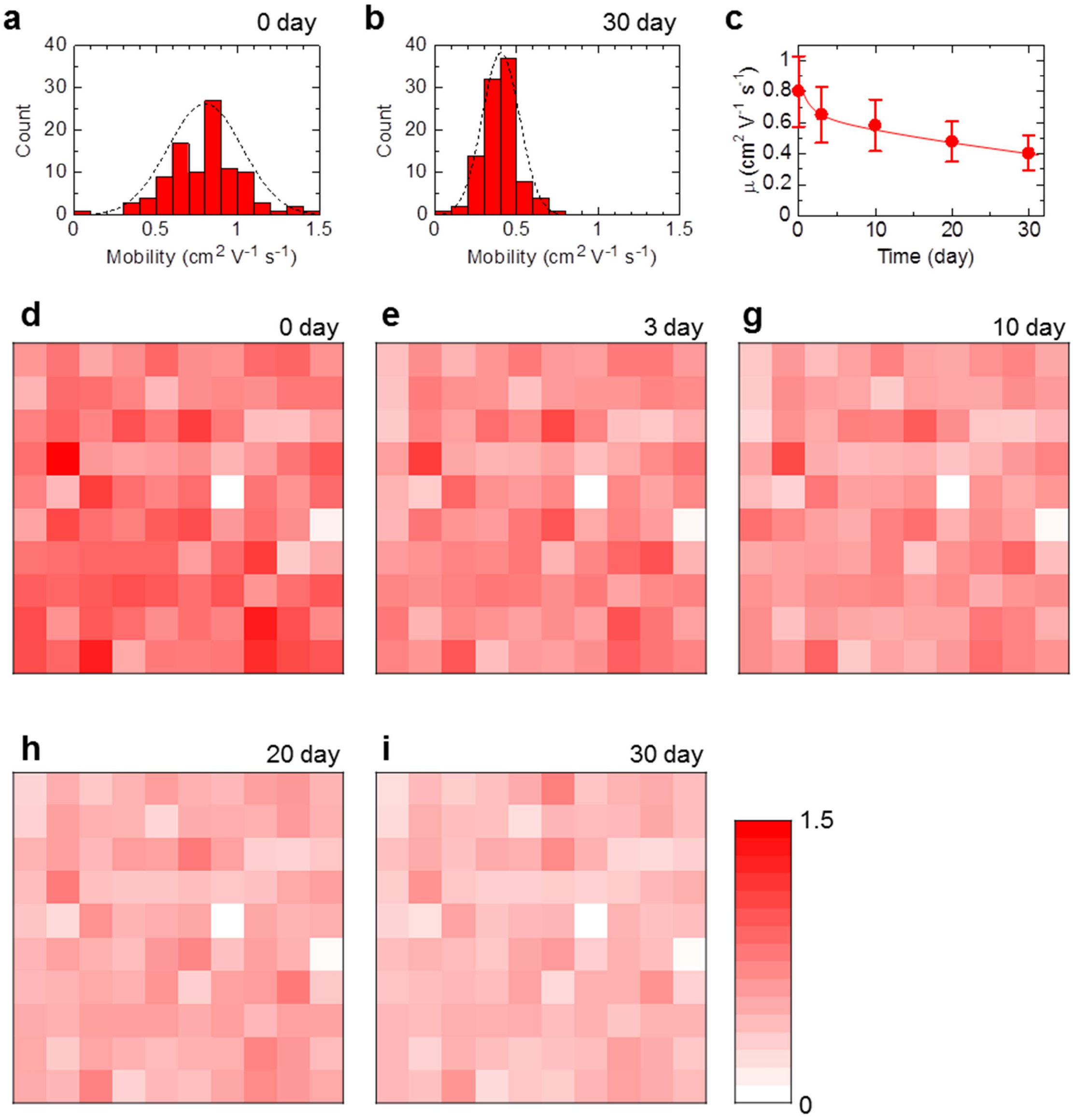

Figure S5. The distribution of mobility values over time.

The distribution of mobility in the array was recorded, **a,** shortly after device fabrication, and, **b,** after 30 days in air. The TFTs functioned well even after 30 days in in an air ambient. The yield of TFT device after 30 days remained at 99%. The average mobility decreased from 0.80 to 0.40 cm^2 V^{-1} s^{-1} and the standard deviation also decreased from 0.23 to 0.11 after 30 days. Although the average mobility degraded, the distribution improved after storage in air. **c,** The mobility as a function of days in air. **d-i,** Two-dimensional plots of mobility variation with color intensity. **d,** Shortly after device fabrication, **e,** after 3 days, **f,** after 10 days, **g,** after 20 days, and **h,** after 30 days.

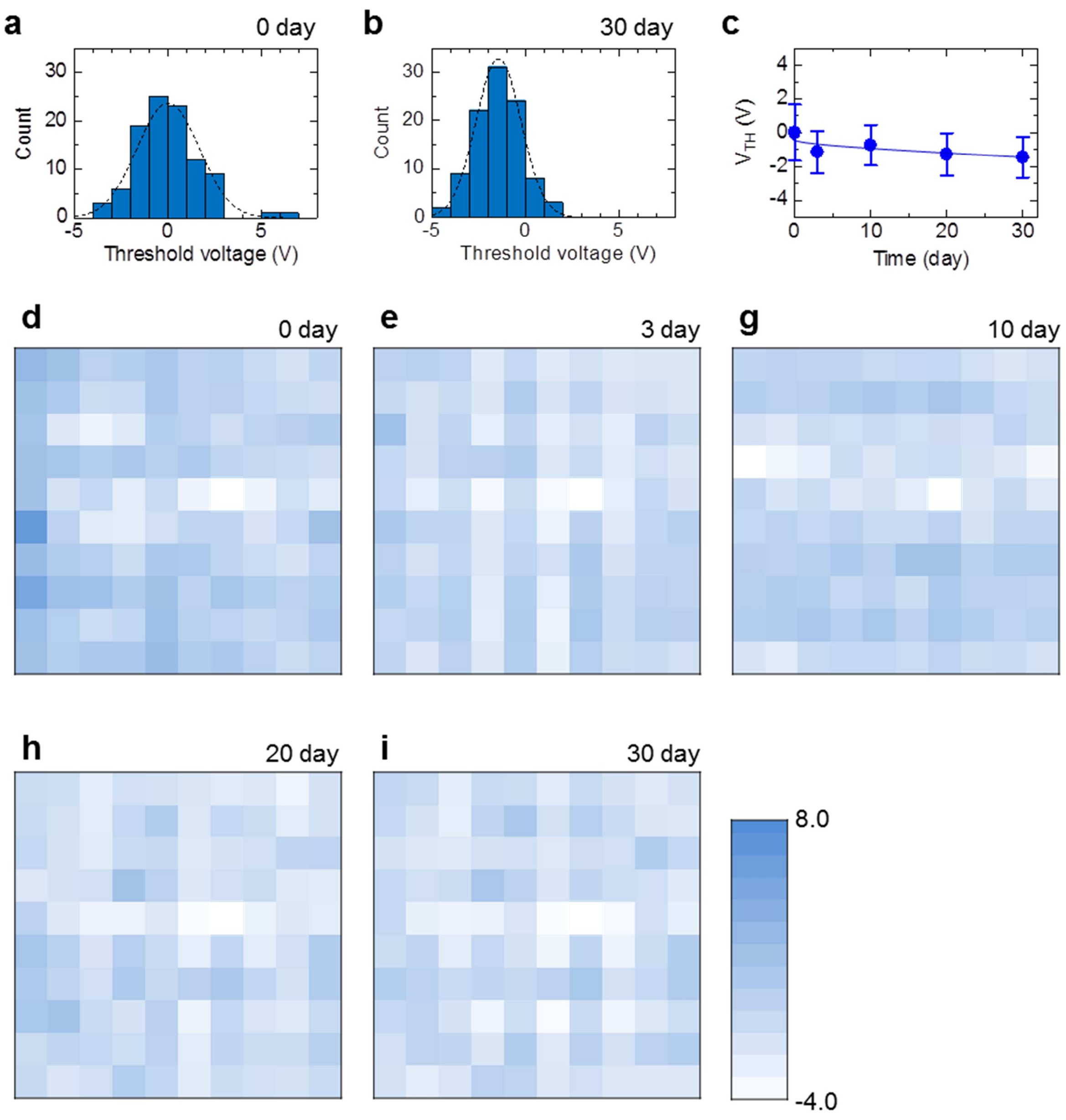

Figure S6. The threshold voltage distribution over time.

The distribution of threshold voltage in the array, **a,** recorded shortly after device fabrication, and, **b,** after 30 days in air. The average threshold voltage shifted from 0.01 to –1.45 V and the standard deviation decreased from 1.77 to 1.20 after 30 days. **c,** The threshold voltage as a function of days in air. **d-i,** Two-dimensional plots of threshold voltage variation with color intensity. **d,** Shortly after device fabrication, **e,** after 3 days, **f,** after 10 days, **g,** after 20 days, and, **h,** after 30 days.

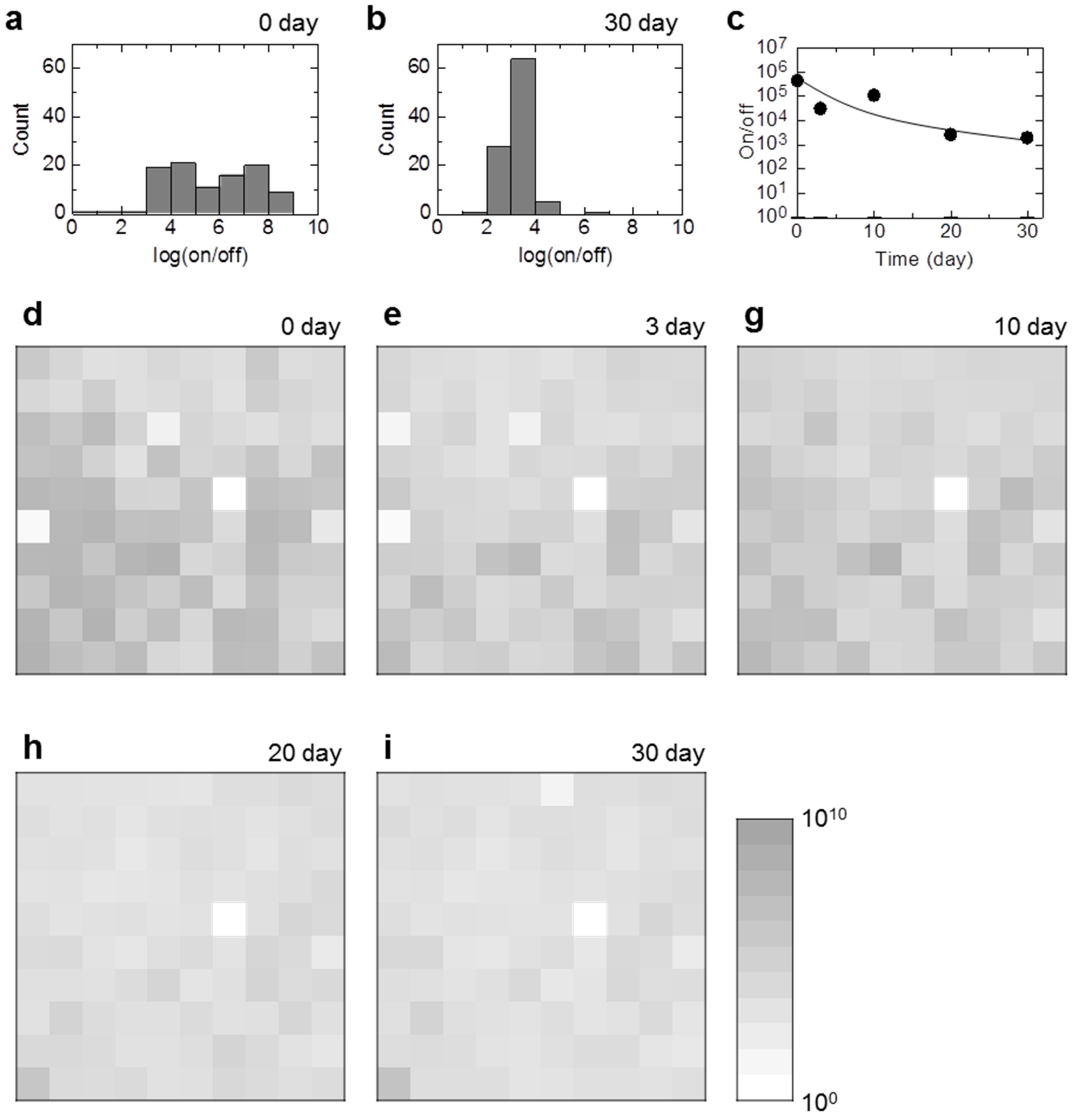

Figure S7. The distribution of on/off ratio over time.

The distribution of on/off ratio in the array recorded, **a,** shortly after device fabrication, and, **b,** after 30 days in air. The average on/off ratio decreased from 4.3 × 10^5 to 1.9 × 10^3 after 30 days. **c,** The on/off ratio as a function of days in air. **d-i,** Two-dimensional plots of on/off ratio variation with color intensity. **d,** Shortly after device fabrication, **e,** after 3 days, **f,** after 10 days, **g,** after 20 days, and **h,** after 30 days.

a

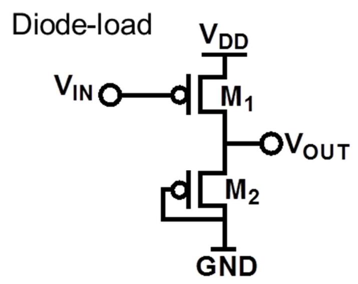

V_{DD} [V]	V_{trip} [V]	Gain
20	17.2	-1.8
15	12.3	-1.7
10	8.0	-1.6

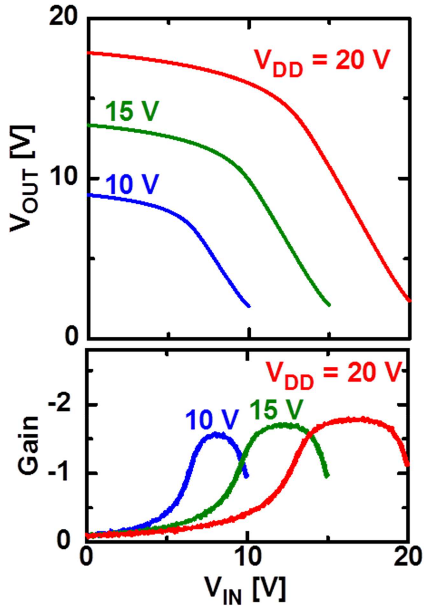

b

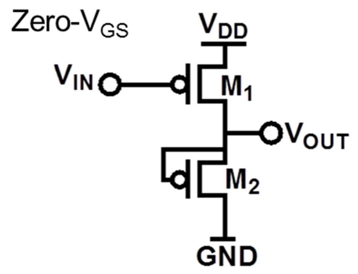

V_{DD} [V]	V_{trip} [V]	Gain
20	12.6	-2.6
15	8.0	-1.9
10	4.1	-1.1

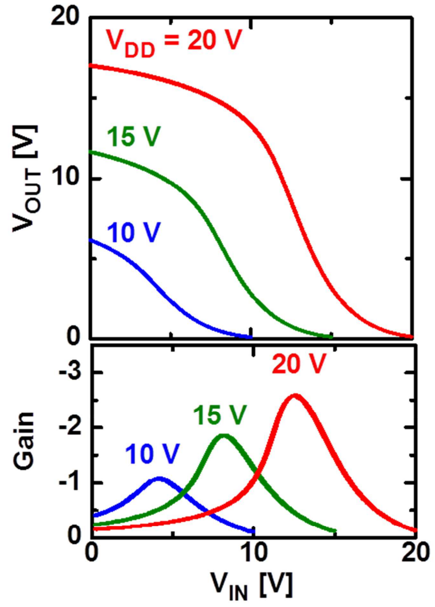

Figure S8. Inverter characteristics.

Fully solution-processed organic TFT integrated circuits were also fabricated on flexible substrates. We fabricated both **a,** a diode-load inverter and **b,** a zero-V_{GS} inverter. Both inverters were functioned well even at an operating voltage of 10 V. Output voltage and signal gain as functions of driving voltage are shown. The trip voltage (V_{trip}), and obtained signal gain are summarized in the inset tables.

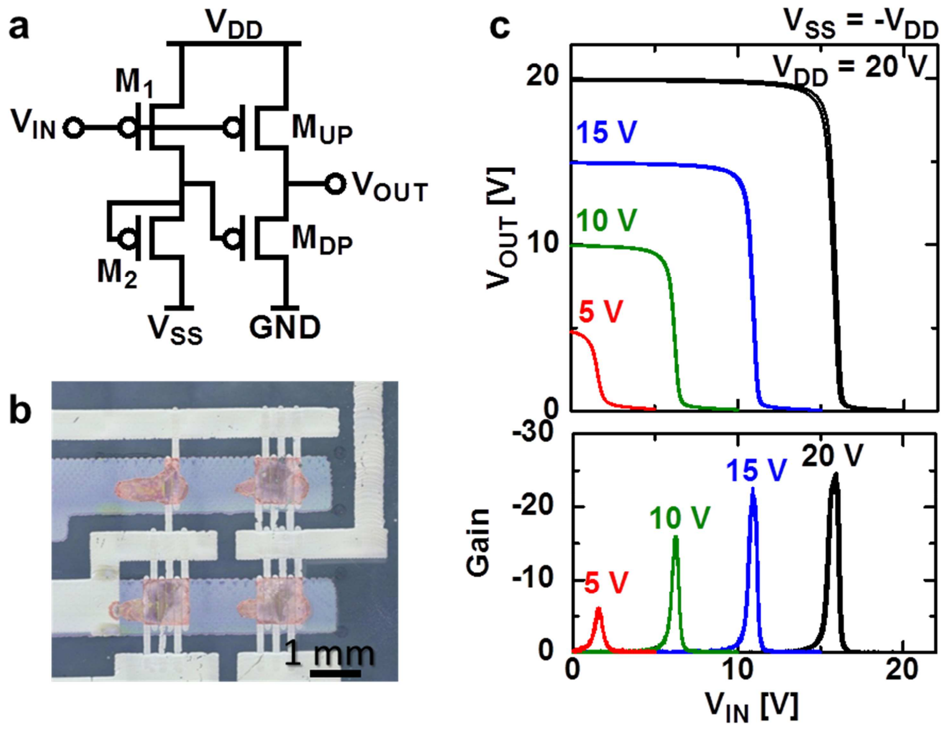

V_{DD} [V]	V_{trip} [V]	Gain
20	16.0	-24
15	10.9	-23
10	6.3	-16
5	1.6	-6.1

Figure S9. Pseudo-CMOS inverter characteristics.

a, Circuit diagram of an organic pseudo-CMOS inverter. This circuit contains only four p-type organic transistors. **b,** The optical photograph of fabricated pseudo-CMOS inverter. **c,** Output voltage and signal gain as functions of driving voltage (*V*DD) with $V_{SS} = -V_{DD}$. The inset table summarizes the trip voltage (V_{trip}) and signal gain at each operating voltage V_{DD}. The pseudo-CMOS inverter functioned well with relatively high signal gain, even at small operating voltages of 5 V.

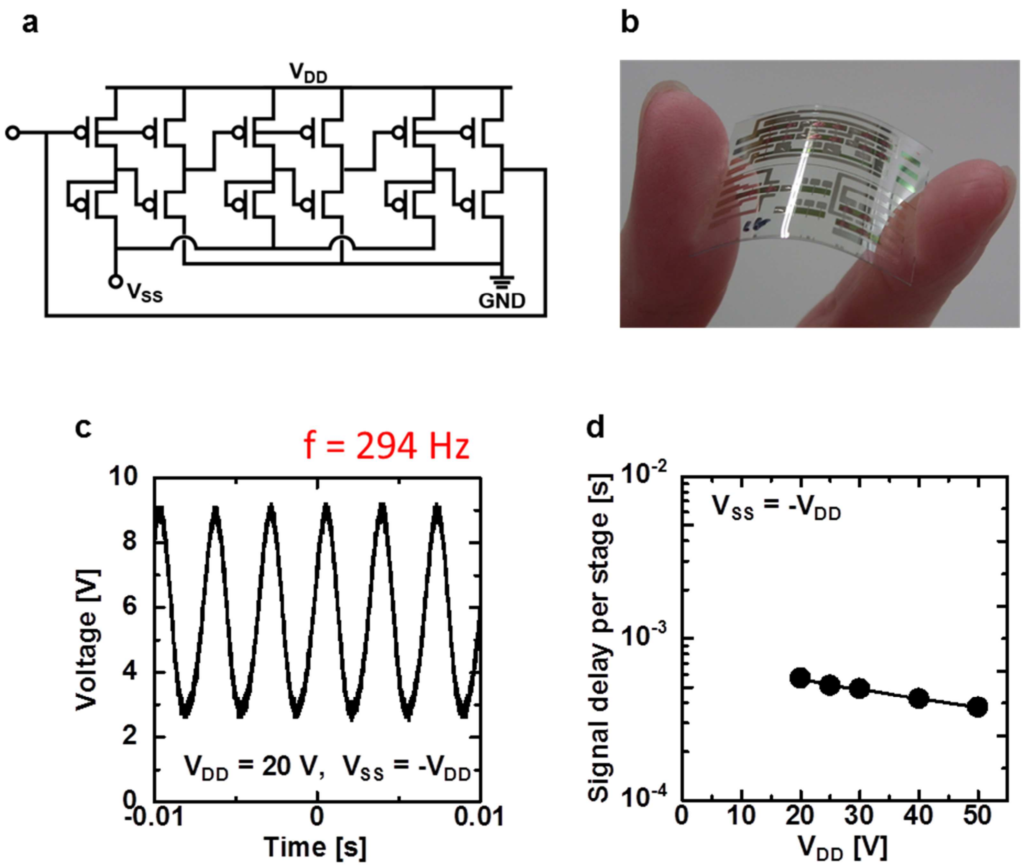

Figure S10. Three-stage pseudo-CMOS ring oscillator characteristics.

a, Circuit diagram for a three-stage organic pseudo-CMOS ring oscillator. **b,** A photograph of the fabricated pseudo-CMOS ring oscillator. **c,** Output with drive voltage (VDD) of 20 V with V_{SS} = –V_{DD}. The obtained frequency was 294 Hz. **d,** Signal propagation delay per stage as a function of driving voltage (V_{DD}) of a three-stage ring oscillator based on pseudo-CMOS inverter with fully-solution processed organic TFTs on flexible PEN substrates. The parameter for calculation of cutt frequency is as follows: channel length (L) of 50 µm, mobility of about 0.5 $cm^2\ V^{-1}s^{-1}$, and the overlap length (Lc) of 700 µm. The estimated signal delay from the cutoff frequency equation $f_c \sim \mu V/[2\pi L(L+Lc)]$ is 234 µs at 20 V and 117 µs at 40 V, which is about three times shorter than the experimental results. The difference between the calculation and the experimental results is caused by the inverter structure (pseudo-CMOS in which four transistors consists one inverter) and other capacitive load.

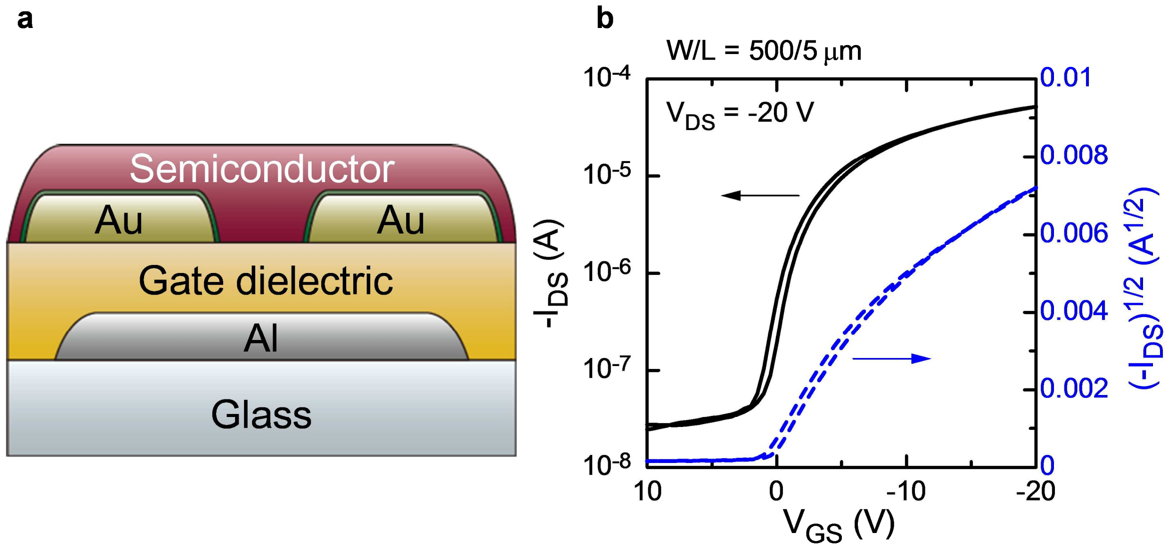

Figure S11. Transistor characteristics with non-printed TFT.

a, A schematic illustration of the TFT. A rigid glass was used as substrate. Evaporated Al and Au were used as gate and source/drain electrodes, respectively. Same gate dielectric layer, contact SAM, and semiconducting layer were used. Semiconducting layer was formed by spin-coating. **b,** A transfer characteristics of non-printed TFT. The channel width and channel length are 500 and 5 μm, respectively. Obtained mobility was 1.7 $cm^2V^{-1}s^{-1}$, on/off ratio was 1.9×10^3, threshold voltage was 0.7 V.
